# Dr. E. T. Wilson

**Published:** 1872-09

**Authors:** Joshua Tucker, Jacob L. Williams, Edward N. Harris

**Affiliations:** Committee.; Committee.; Committee.


### OBITUARY.

Dr. E. T. Wilson.—At a special meeting of the American Academy of Dental Science, convened in Boston, July 9th 1872, the following resolutions were adopted:

Whereas, it has pleased Almighty God to remove from among us, Elisha T. Wilson, M. D., the first president and late vice-president of the American Academy of Dental Science; it is therefore

*Resolved*, That this Academy has received with sincere sorrow the painful intelligence of the decease of our highly esteemed friend and associate.

*Resolved*, That by the death of Dr. Wilson, the profession of dentistry has lost one of its most eminent practitioners, Boston one of its most useful citizens, and our Academy one of its most valued and energetic members, and that we shall always retain the warmest recollections of his many excellencies of character, his kindness of heart, and his generous hospitality.

*Resolved*, That a copy of these resolutions be presented to the family of the deceased, and that we tender to them our deep sympathy in their great bereavement and sorrow.

Also, that these resolutions be published in the American Journal of Dental Science, and the *Dental Cosmos*.

Joshua Tucker.
Jacob L. Williams. } *Committee.*
Edward N. Harris.

